# SRT1720‐induced activation of SIRT1 alleviates vascular smooth muscle cell senescence through PKA‐dependent phosphorylation of AMPKα at Ser485

**DOI:** 10.1002/2211-5463.12895

**Published:** 2020-06-05

**Authors:** Jin Young Sung, Seul Gi Kim, Du Hyong Cho, Jae‐Ryong Kim, Hyoung Chul Choi

**Affiliations:** ^1^ Department of Pharmacology College of Medicine Yeungnam University Daegu Korea; ^2^ Smart‐aging Convergence Research Center College of Medicine Yeungnam University Daegu Korea; ^3^ Department of Biochemistry and Molecular Biology College of Medicine Yeungnam University Daegu Korea

**Keywords:** p‐AMPK (Ser485), SIRT1, SRT1720, telomere length, VSMC senescence

## Abstract

Aging is a major risk factor for hypertension and atherosclerosis, and vascular smooth muscle cell (VSMC) senescence can promote aging‐related vascular diseases. Sirtuin‐1 (SIRT1) and AMP‐activated protein kinase (AMPK) were previously reported to modulate vascular senescence; however, its effects have not been well characterized. To determine the nature of the interaction between SIRT1 and AMPK in VSMC senescence, we investigated the effects of SRT1720 on its downstream targets of SIRT1 and the phosphorylation of AMPKα at Ser485. During Adriamycin‐induced VSMC senescence, SRT1720 increased the activity of SIRT1 and AMPKα phosphorylation at Ser485 via the cAMP–protein kinase A (PKA) pathway. Telomere length and telomerase reverse transcriptase expression were increased by SIRT1 activation with SRT1720. Taken together, these data show that activation of the SIRT1/cAMP–PKA/p‐AMPKα (Ser485) pathway may be an effective antisenescence mechanism for VSMCs.

## Graphical abstract

Sirtuin‐1 (SIRT1) and AMP‐activated protein kinase (AMPK) were reported to modulate vascular senescence. We investigated the effects of SRT1720 on downstream targets of SIRT1 and the phosphorylation of AMPKα at Ser485. During Adriamycin‐induced vascular smooth muscle cell senescence, SRT1720 increased the activity of SIRT1 and AMPKα phosphorylation at Ser485 via the cAMP–protein kinase A (PKA) pathway and telomerase reverse transcriptase expression. Activation of the SIRT1/cAMP–PKA/p‐AMPKα (Ser485) pathway may be an effective antisenescence mechanism for vascular smooth muscle cell.

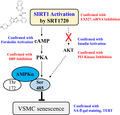

**Abbreviations**
ADR: Adriamycin
AMPK: AMP‐activated protein kinase
DAB: 2,4‐diaminobutyric acid
PKA: protein kinase A
SA‐β‐gal: senescence‐associated β‐galactosidase
SIRT1: sirtuin‐1
TERT: telomerase reverse transcriptase
VSMC: vascular smooth muscle cell

## Introduction

Cellular senescence is a permanent cell growth arrest state and is associated with a series of morphological and physiological changes, such as in biomarkers of cellular senescence and shortening of telomeres [1]. Vascular smooth muscle cell (VSMC) senescence contributes to cardiovascular diseases, such as atherosclerosis and vascular calcification [2, 3]. Although VSMC senescence has been extensively examined, its underlying molecular mechanisms remain elusive.

Metabolic regulators play important roles in the control of VSMC senescence, and sirtuin‐1 (SIRT1) and AMP‐activated protein kinase (AMPK) act as energy sensors that regulate cell proliferation, survival and senescence [4]. SIRT1 has attracted considerable attention as a mediator of health and longevity in response to calorie restriction [5], and recent studies have shown that SIRT1 plays a protective role against cardiovascular disease by defending against oxidative stress and delaying cellular senescence [6, 7]. SIRT1 has also been reported to protect against aortic stiffness [8] and to reduce DNA damage, apoptosis and VSMC senescence [9]. SRT1720, a specific activator of SIRT1, is one of the pharmaceutical compounds that are being investigated for aged‐related disease in the treatment of metabolic diseases associated with aging *in vitro* [10, 11, 12]. SRT1720 extends the lifespan of healthy mice and ameliorates the senescence of endothelial cells [13, 14]. However, the mechanisms of SRT1720 in the senescent VSMCs remain unknown.

The AMPK signaling pathway controls the aging process via an integrated signaling network and different phosphorylation sites. AMPK is generally activated by phosphorylation in the presence of an elevated AMP/ATP ratio at Thr172 [15], and phosphorylation of AMPK at Ser485 has been identified as an autophosphorylation site [16] that is targeted by cAMP–protein kinase A (PKA) [17] and AKT pathways [18]. Phosphorylation of AMPK at Ser485 by AKT in response to insulin stimulation is probably involved in the insulin‐regulated inhibition of AMPK activity [19], and phosphorylation of AMPK at Ser485 by PKA alters accessibility of AMPK phosphorylation at Thr172 and its expression [20]. However, the basic level of AMPK activity and its responses to different upstream stimuli can be different in cellular senescence. In addition, the roles played by each regulation of AMPK phosphorylation sites in VSMC senescence have not been fully clarified.

Crosstalk between SIRT1 and AMPK signaling pathways is believed to regulate cellular senescence of mammals [4], but it has not been determined whether interactions between SIRT1 and AMPK signaling pathways offer a potential means of controlling VSMC senescence. Therefore, in this study, we investigated the role of SIRT1 and AMPK signaling pathways in the alleviation of Adriamycin (ADR)‐induced VSMC senescence using SRT1720.

## Materials and methods

### Reagents and antibodies

SRT1720 was supplied by Santa Cruz Biotechnology, Inc. (Santa Cruz, CA, USA). Doxorubicin (ADR), EX527, wortmannin, insulin, H‐89 and forskolin were supplied by Sigma (St. Louis, MO, USA). Compound C was obtained from Calbiochem (La Jolla, CA, USA). Antibodies were purchased from the following vendors: SIRT1, p‐AMPK (Ser485), H3, p‐AKT, AKT p‐PKA (Thr197) and PKA (Cell Signaling Technology, Danvers, MA, USA); and Ac‐H3, p53, p21, p16, telomerase reverse transcriptase (TERT), SMP‐30 and β‐actin (Santa Cruz Biotechnology).

### Cell culture and western blot analysis

Rat aortic VSMCs from passages 4–8 were seeded and cultured in high‐glucose Dulbecco’s modified Eagle’s medium supplemented with 10% FBS (HyClone, Logan, UT, USA) and 50 U·mL^−1^ penicillin. Cells were lysed in RIPA lysis buffer and incubated on ice for 10 min followed by centrifugation at 13 000 ***g*** for 20 min at 4 °C. Protein concentration was determined from centrifuged supernatant by using a Bradford assay (Bio‐Rad, Hercules, CA, USA). For western blotting, proteins were separated by SDS/PAGE and transferred to poly(vinylidene difluoride) membranes, which were immunoblotted with the indicated primary antibodies and then with corresponding secondary antibodies (1 : 5000). Signals were visualized using chemiluminescence detection reagents (Millipore, Billerica, MA, USA), according to the manufacturer’s instructions.

### SIRT1 activity assay

SIRT1 activity assay was performed using a commercial fluorogenic SIRT1 Assay Kit (BPS Bioscience, San Diego, CA, USA). The fluorophore was excited at 350 nm and detected at 460 nm on a fluorometric plate reader (Bio‐Rad).

### Immunohistochemical staining

VSMC was performed immunohistochemical staining after cell seeding on six‐well plates. Following washing, cells were blocked by blocking buffer for 1 h, prior to incubation with primary antibodies against SIRT1 at a dilution of 1 : 50 overnight at 4 °C. After washes with PBS‐T, cells were incubated with HRP–labeled secondary antibody (1 : 2000) for 1 h at room temperature. After washing, the bound complexes were visualized using the application of a solution of 2,4‐diaminobutyric acid (DAB) kit (Thermo Scientific, Waltham, MA, USA).

### Senescence‐associated β‐galactosidase staining

VSMCs were seeded on six‐well plates and fixed with 4% formaldehyde for 30 min at room temperature. Cells were then washed with PBS and followed by senescence‐associated β‐galactosidase (SA‐β‐gal) staining kit (Cell Signaling Technology). The percentage of blue cells per 100 cells observed under a light microscope was determined.

### Immunofluorescence analysis

VSMCs were fixed with 4% buffered paraformaldehyde for 30 min and permeabilized with 0.2% Triton X‐100 for 5 min at room temperature. Cells were then blocked with 5% normal goat serum in PBS‐T followed by incubation with anti‐SIRT1, p‐AMPK (Ser485) and p‐PKA (1 : 100) with incubation overnight at 4 °C. Cells were treated with secondary Alexa Fluor 488 goat rabbit serum and 546 goat mouse serum at 1 : 500 (Invitrogen, Carlsbad, CA, USA) with incubation for 45 min at room temperature. The nuclei were stained with 4′,6‐diamidino‐2‐phenylindole for 10 min at room temperature. Imaging of cells that were fixed and stained by immunofluorescence was performed using Olympus microscope with LMPLFLNM Plan FL 20× lenses (Tokyo, Japan).

### Transfection of siRNA and plasmid DNA

VSMCs were transfected with control siRNA and SIRT1 siRNA by using Lipofectamine 2000 reagent (Invitrogen), according to the manufacturer’s instructions. Cells were resuspended in complete Dulbecco’s modified Eagle’s medium and incubated for 24–48 h. Flag‐SIRT1 was obtained from Addgene plasmid repository (Addgene plasmid 1791; Addgene, Watertown, MA, USA; ME Greenberg, Harvard Medical School, Boston, MA, USA). For transfection, VSMCs were seeded on six‐well plates. When cells were approximately 80% confluent, cells were transfected in Opti‐MEM for 24 h with Lipofectamine 2000 reagent mixture containing SIRT1 plasmid DNA.

### Quantitative real‐time RT‐PCR

Extraction of genomic DNA of cells was performed as with the DNA extraction kit (BioSewoom, Daejeon, Korea), following the manufacturer’s instruction, and dissolved in TE buffer (pH 8.0). Telomere lengths were determined by quantitative real‐time RT‐PCR as previously described [21]. Quantitative PCR was performed using the SYBR^®^ Select Master Mix (Enzynomics, Seoul, Korea) with a Bio‐Rad real‐time system. The parameters were as follows: 95 °C for 10 s, followed by 40 cycles of 60 °C for 15 s and 72 °C for 15 s. Primers used were as follows: Tel‐F 5′ GGT TTT TGA GGG TGA GGG TGA GGG TGA GGG TGA GGG t‐3′, and Tel‐R 5′‐TCC CGA CTA TCC CTA TCC CTA TCC CTA TCC CTA TCC CTA‐3′; AT1 rat‐F 5′‐ACG TGT TCT CAG CAT CGA CCG CTA CC‐3′ and AT1 rat‐R 5′‐AGA ATG ATA AGG AAA GGG AAC AAG AAG CCC‐3′ (Bioneer, Daejeon, Korea). The quantities of telomeric DNA were normalized to that of AT1 [22].

### Statistical analysis

Results in the bar graph are expressed as means ± SEM for three independent experiments. Statistical analysis was performed with Student’s *t*‐test and ANOVA followed by Bonferroni’s *post hoc* test for multiple‐group comparisons using graphpad prism 5.0 (GraphPad Software, San Diego, CA, USA). The *P*‐values <0.05 were considered significant.

## Results

### Pharmacological activation of SIRT1 increases the expression level of p‐AMPK (Ser485) through the cAMP–PKA pathway in VSMCs

**Fig. 1:**
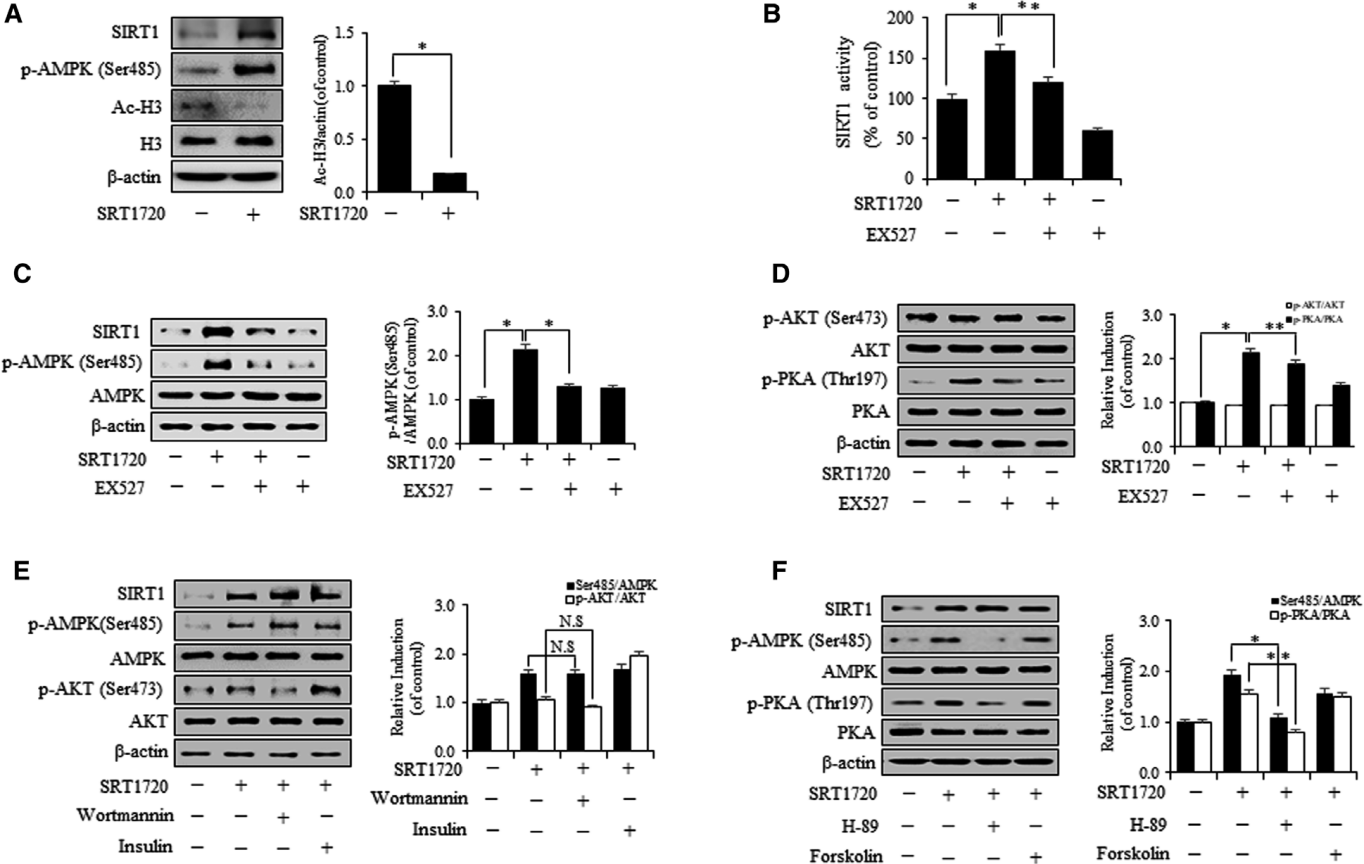
SRT1720 increases p‐AMPK (Ser485) via p‐PKA, but not p‐AKT, in VSMC. (A) VSMC was stimulated with SRT1720 (20 µm) for 2 h. (B) VSMC was pretreated with EX527 (10 µm) for 1 h and then incubated with SRT1720. Intracellular SIRT1 activity was measured using a fluorogenic SIRT1 assay kit. (C, D) VSMC was pretreated with EX527 and then incubated with SRT1720, and protein levels were analyzed using western blot. (E) VSMC was treated with or without wortmannin (200 nm) and insulin (100 nm) for 45 min, and then treated with SRT1720. (F) VSMC was treated with or without H‐89 (10 µm) for 1 h and forskolin (10 µm) for 30 min and then treated with SRT1720. Results are expressed as means ± SEM and are representative of three independent experiments. Statistical analysis was performed with Student’s *t*‐test and ANOVA followed by Bonferroni’s *post hoc* test. *P < 0.05, **P < 0.01. N.S, not significant.

To identify a role of SIRT1 activator, we first tested the effects of SRT1720 on SIRT1 and p‐AMPK (Ser485). As expected, SRT1720 increased the activity of SIRT1 and the expression level of p‐AMPK (Ser485) as compared with nontreated controls (Fig. 1A,B). Furthermore, the acetylation of H3 was reduced by SRT1720 (Fig. 1A). AMPK at Ser485 is known to be phosphorylated by the downstream kinases of AKT [18] and PKA [23]. To determine whether these molecules act as upstream kinase for p‐AMPK (Ser485), we experimented using respective activators and inhibitors by treatment with SRT1720 and EX527 (Fig. 1C). The expression level of p‐PKA was upregulated by SRT1720, but neither SRT1720 nor EX527 changed the expression level of p‐AKT (Fig. 1D). In addition, to investigate whether the phosphoinositide 3‐kinase (PI3K)/AKT pathway contributes to AMPK phosphorylation at Ser485, wortmannin and insulin, which are a specific inhibitor of PI3K and activator of AKT, respectively, were used. As shown in Fig. 1E,F, despite treatment with SRT1720, the expression level of p‐AMPK (Ser485) was unchanged by wortmannin or insulin. However, H‐89 and forskolin, which are a specific inhibitor of PKA and activator of adenylyl cyclase, respectively, modestly changed the expression level of p‐AMPK (Ser485). These findings indicate that SRT1720 regulates the expression level of p‐AMPK (Ser485) via a p‐PKA pathway in VSMCs.

### Inhibition of SIRT1 decreases the effect of SRT1720 in ADR‐induced VSMC senescence

**Fig. 2:**
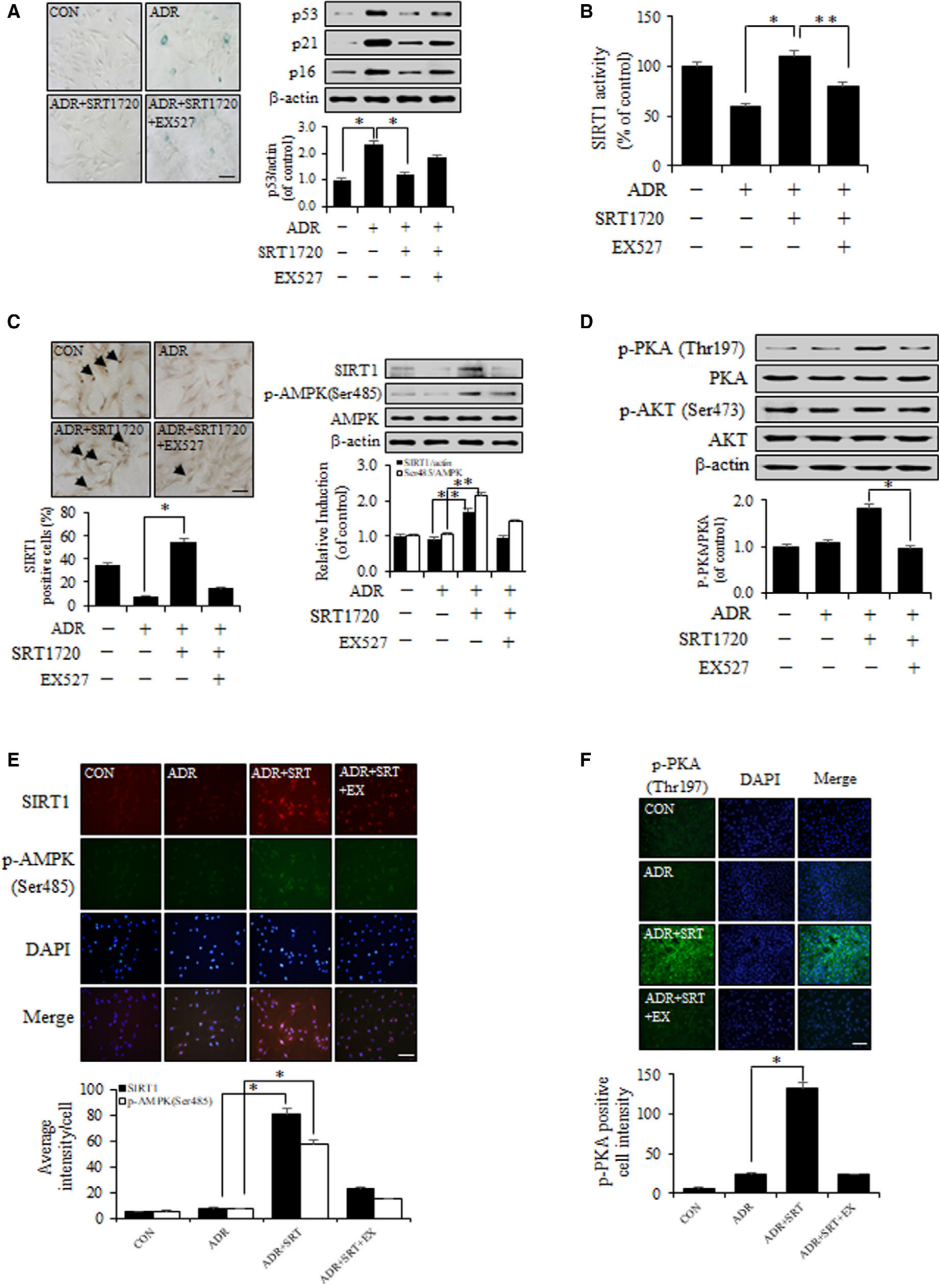
SRT1720 ameliorates ADR‐induced VSMC senescence via a cAMP–PKA/p‐AMPK (Ser485) pathway. (A) VSMC was stimulated with ADR (500 nm) for 4 h and cotreated with EX527 or SRT1720. After incubation, SA‐β*‐*gal staining and protein levels were performed. The images were taken at an original magnification ×200. (B) Intracellular SIRT1 activity was measured using a fluorogenic SIRT1 assay kit. (C) DAB staining was performed using antibodies against SIRT1 (1 : 50). Immunospecific staining is shown in brown (SIRT1). The percentage of brown cells per 100 cells observed under a light microscope was determined. The images were taken at an original magnification ×200. (D) Protein levels were analyzed by western blot. (E, F) Phenotype of cultured VSMC was identified by indirect immunofluorescent staining using antibodies for SIRT1, p‐AMPK (Ser485) and p‐PKA (1 : 100). The images were taken at an original magnification ×200. Results are expressed as means ± SEM and are representative of three independent experiments. Statistical analysis was performed with Student's *t*‐test and ANOVA followed by Bonferroni's *post hoc* test. *P < 0.05, **P < 0.01. Scale bars, 200 µm.

ADR provides more efficient means of generating cellular senescence than replicative cellular senescence [24]. In this study, ADR increased prosenescent protein levels and SA‐β‐gal activity, but SRT1720 recovered ADR‐induced VSMC senescence (Fig. 2A). EX527 also substantially inhibited SIRT1 activity. SRT1720 increased SIRT1 deacetylase activity in ADR‐induced VSMC senescence, but EX527 decreased (Fig. 2B). To determine the effect of p‐AMPK (Ser485) on SIRT1 activity, we performed immunohistochemical staining after treating SRT1720 and EX527 to ADR‐induced VSMC senescence. The expression level of SIRT1 was diminished by EX527 in DAB staining and western blot analysis. Furthermore, the expression levels of SIRT1 and p‐AMPK (Ser485) were increased by SRT1720 in ADR‐induced VSMC senescence but decreased by EX527 (Fig. 2C). Interestingly, the expression level of p‐AKT was not changed despite treating with SRT1720, but the expression level of p‐PKA was similar to that of SIRT1 and p‐AMPK (Ser485) (Fig. 2D). Microscopic examinations revealed that SIRT1 and p‐AMPK (Ser485) were deficient and non‐localized in ADR‐treated VSMCs, and that their expression levels were increased by SRT1720, but not by EX527 (Fig. 2E). The expression level of p‐PKA also was increased by SRT1720, but not by EX527, in ADR‐treated VSMCs (Fig. 2F). These observations suggest that SIRT1 protects VSMCs from ADR‐induced cellular senescence via the cAMP–PKA/p‐AMPK (Ser485) pathway.

### Knockdown of SIRT1 diminishes the effect of SRT1720, and the overexpression of SIRT1 alleviates ADR‐induced VSMC senescence via a cAMP–PKA/p‐AMPK (Ser485) pathway

**Fig. 3:**
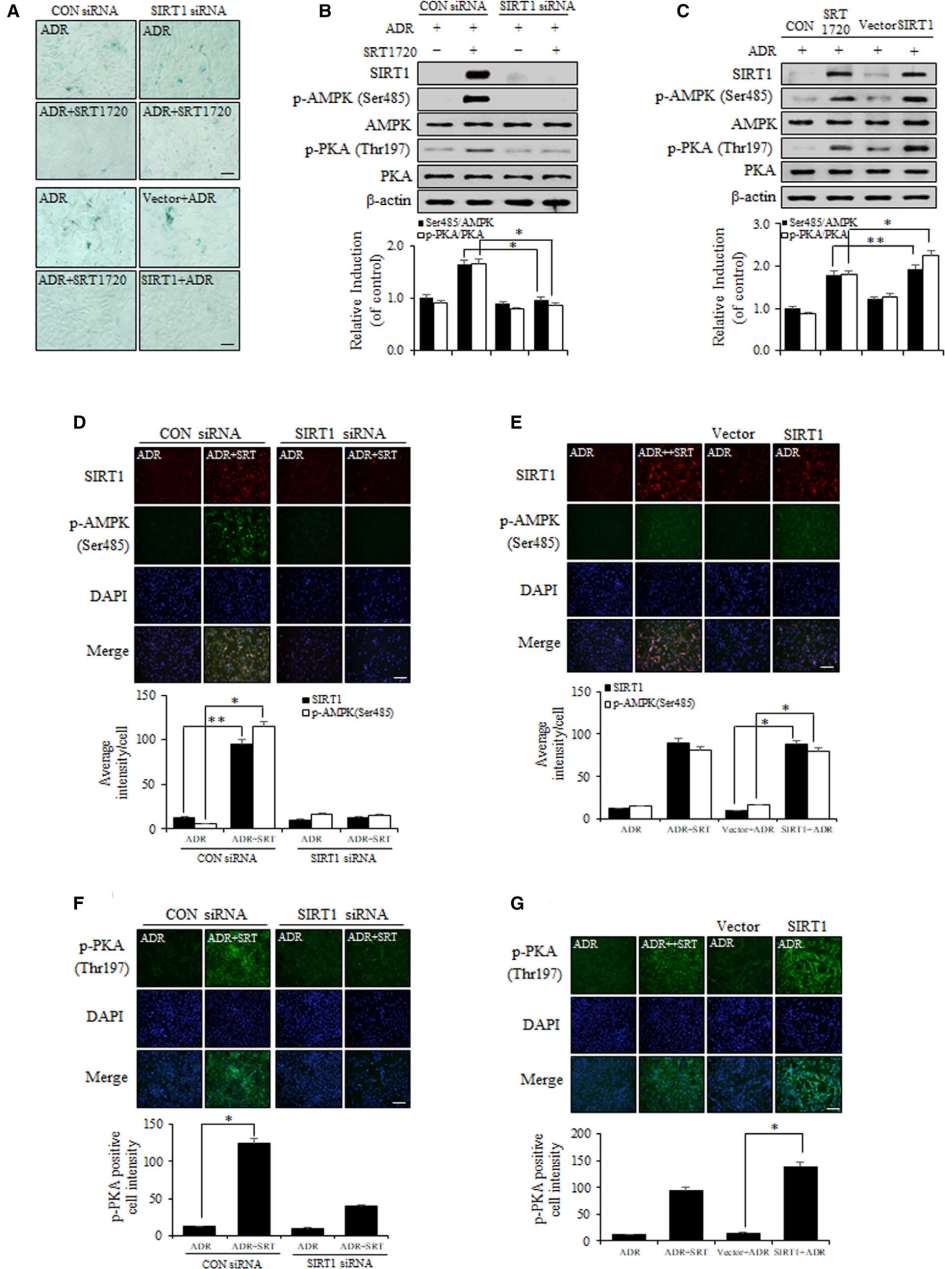
Knockdown of SIRT1 decreases the antiaging effect of SRT1720, and overexpression of SIRT1 alleviates ADR‐induced VSMC senescence via a cAMP–PKA/p‐AMPK (Ser485) pathway. (A) VSMC was transfected with control or SIRT1 siRNA (10 µm) for 48 h and stimulated with ADR. VSMC was transduced with Xp‐His vector or flag‐SIRT1 plasmid to overexpress SIRT1 for 24 h. After infection, cells were stained with SA‐β‐gal. The images were taken at an original magnification ×200. (B, C) Protein levels were analyzed using western blot. (D–G) VSMC was transfected with control or SIRT1 siRNA and transduced with vector or flag‐SIRT1 plasmid to overexpress SIRT1, respectively. Phenotype of cultured VSMC was identified by indirect immunofluorescent staining using antibodies for SIRT1, p‐AMPK (Ser485) and p‐PKA (1 : 100). Images were taken at an original magnification ×200. Results are expressed as means ± SEM and are representative of three independent experiments. Statistical analysis was performed with Student's *t*‐test and ANOVA followed by Bonferroni's *post hoc* test. *P < 0.05, **P < 0.01. Scale bars, 200 µm.

To determine the antiaging effect of SRT1720 on ADR‐induced VSMC senescence, we examined the effects of knockdown and overexpression of SIRT1. SA‐β‐gal activity was greater in cells transfected with SIRT1 siRNA than with control siRNA despite SRT1720 treatment. In contrast, SA‐β‐gal activity was lower in SIRT1‐overexpressing cells than in control vector‐transfected cells in the presence of ADR (Fig. 3A). We then investigated whether the effect of SIRT1 on ADR‐induced VSMC senescence was dependent on the cAMP–PKA/p‐AMPK (Ser485) pathway. The expression levels of SIRT1 and p‐AMPK (Ser485) were increased by SRT1720 in ADR‐treated VSMCs transfected with control siRNA. In addition, the overexpression of SIRT1 significantly increased the expression levels of SIRT1 and p‐AMPK (Ser485) as compared with control vector‐transfected cells in ADR‐treated VSMCs. Furthermore, the expression level of p‐PKA was similar to that of SIRT1 and p‐AMPK (Ser485) after knockdown or overexpression of SIRT1 (Fig. 3B,C). Microscopic examinations revealed that the abundances and colocalizations of SIRT1 and p‐AMPK (Ser485) were increased by SRT1720 in ADR‐treated VSMCs after control siRNA transfection, but not after SIRT1 siRNA transfection (Fig. 3D). In contrast with the results obtained for SIRT1 siRNA‐transfected VSMCs, the overexpression of SIRT1 increased the expression levels of SIRT1 and p‐AMPK (Ser485) as compared with control vector‐transfected cells as determined by immunofluorescence despite ADR treatment (Fig. 3E). The expression level of p‐PKA also was similar to that of SIRT1 and p‐AMPK (Ser485) after SIRT1 knockdown or overexpression in immunofluorescence analysis (Fig. 3F,G). These findings suggest that SIRT1 is an essential mediator of the cAMP–PKA/p‐AMPK (Ser485) pathway, which protects VSMCs from ADR‐induced cellular senescence.

### SIRT1 increases telomere lengths and the expression level of TERT in ADR‐treated VSMCs

**Fig. 4:**
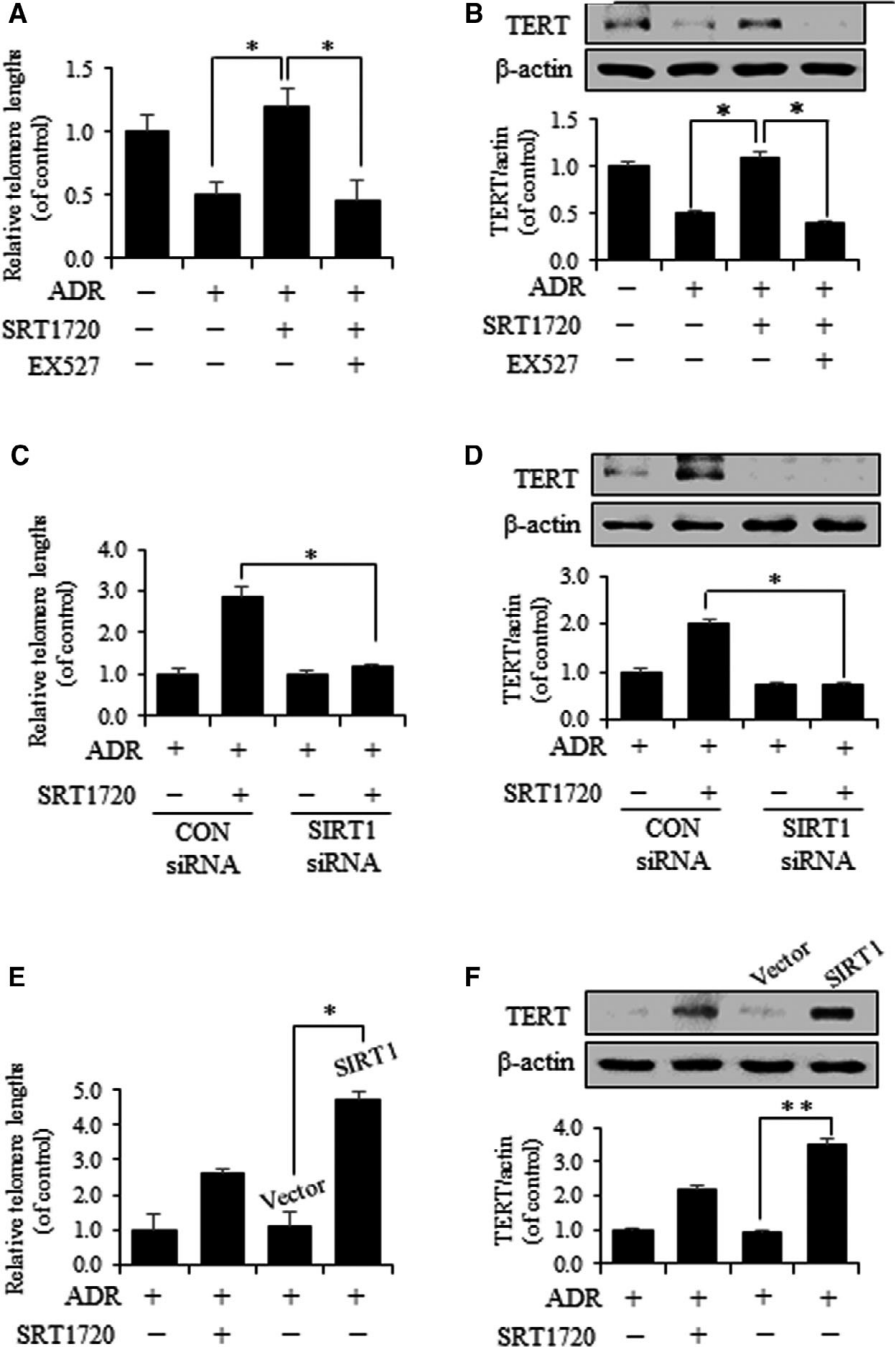
SRT1720 increases telomere lengths and TERT expression in senescent VSMC. (A) VSMC was stimulated with ADR and then treated with EX527 or SRT1720. Relative telomere lengths were detected by qPCR. (B) The expression level of TERT was assessed by western blot. (C) VSMC was transfected with control or SIRT1 siRNA (10 µm) and stimulated with ADR and then treated with SRT1720, and relative telomere lengths were determined by qPCR. (D) The expression level of TERT was assessed by western blot. (E) VSMC was transduced with vector or flag‐SIRT1 plasmid to overexpress SIRT1 and then stimulated with ADR and treated with SRT1720. Relative telomere lengths were determined by qPCR. (F) The expression level of TERT was assessed by western blot. Results are expressed as means ± SEM and are representative of three independent experiments. Statistical analysis was performed with Student’s *t*‐test and ANOVA followed by Bonferroni’s *post hoc* test. *P < 0.05, **P < 0.01.

Telomere shortening has been shown in several cell types during aging [25], and SIRT1 is closely related to a positive regulator of telomere length *in vivo* and attenuates age‐associated telomere shortening [26]. To investigate the effects of SIRT1 on telomere lengths and TERT protein in VSMC senescence, we treated SRT1720 in the presence of ADR. ADR‐treated VSMC had shorter telomere lengths than SRT1720‐treated cell, and telomere lengths also were shortened by EX527‐treated cell (Fig. 4A). In addition, we found that the expression level of TERT was decreased in ADR‐induced VSMC senescence but increased in SRT1720‐treated cells (Fig. 4B). Telomere lengths also were shortened in cells pretreated with SIRT1 siRNA (Fig. 4C) but were prolonged in cells treated with SIRT1 vector regardless of ADR treatment (Fig. 4E). Consistently, the expression level of TERT was reduced by pretreatment with SIRT1 siRNA as compared with control siRNA in senescent cells (Fig. 4D). In contrast, the expression level of TERT was higher in ADR‐treated VSMCs overexpressing SIRT1 than in vector‐transduced VSMCs (Fig. 4F). These findings show that SIRT1 increases telomere lengths and the expression level of telomerase in ADR‐treated VSMCs.

## Discussion

This study demonstrated that SRT1720 inhibited VSMC senescence through the SIRT1 and p‐AMPK (Ser485) signaling pathway. The antiaging effect of SRT1720 on senescent VSMCs was demonstrated based on the following results: (a) increased expression levels of SIRT1, p‐AMPK (Ser485) and p‐PKA; (b) decreased SA‐β‐gal activity; (c) reduced expression levels of p53, p21 and p16 proteins; and (d) reversed telomere lengths and expression level of TERT.

Aging is the most important determinant of vascular health and progressively leads to morphological and functional changes in vascular walls associated with cardiovascular diseases, such as hypertension and atherosclerosis [3]. VSMCs are known to be intimately involved in age‐associated changes in the vasculature. VSMC senescence reduces vascular contractility, which maintains vascular tone, and also alters vascular physiology and pathophysiology [27]. This study shows that SIRT1 and AMPK act as novel molecular links that retard VSMC senescence. The major findings of this study are as follows. Firstly, the expression and activity of SIRT1 significantly increased the expression levels of p‐AMPK (Ser485) and p‐PKA (Fig. 1). Secondly, SRT1720 treatment in ADR‐induced VSMC senescence substantially downregulated the age‐associated phenotypic changes (p53, p21 and p16) (Fig. 2). Thirdly, genetic upregulation of SIRT1 decreased ADR‐induced VSMC senescence. In experiments that use SIRT1 DNA vector to overexpress SIRT1, it serves as an essential mediator of the cAMP–PKA/p‐AMPK (Ser485) pathway, which acts to protect against ADR‐induced VSMC senescence (Fig. 3). Lastly, SIRT1 increased telomere lengths and the expression level of TERT in ADR‐treated VSMCs (Fig. 4), which is in line with previous reports regarding the relationship between SIRT1 and telomere lengths [28]. It has also been reported that VSMC senescence and telomere shortening are positively related to atherosclerotic plaque formation on normal vessel walls [29].

The increased SIRT1 activity by pharmacological intervention has been found to slow the onset of aging and delay age‐associated diseases. The previous study showed that the overexpression of SIRT1 and use of resveratrol for increasing SIRT1 activity and expression level improved health and extended the lifespan of mice [30]. SRT1720 also improved survival and health of mice [11] and has been reported to activate SIRT1 more than resveratrol [10]. In addition, a previous study showed that reduced SIRT1 expression level by high glucose is reversed by SRT1720 [31]. However, the effect of SRT1720 on VSMC senescence has not been fully elucidated. Therefore, this study revealed the involvement of SIRT1‐dependent mechanisms by SRT1720 on VSMC senescence.

The previous studies also have demonstrated that AMPK at Ser485 is phosphorylated by upstream kinases such as protein kinase B/AKT [16] in response to insulin stimulation. In addition, crosstalk between SIRT1 and AKT has been well documented, and SIRT1 has been reported to attenuate aging through the PI3K/AKT pathway in several cell lines [32]. However, in this study, wortmannin or insulin did not alter the expression level of p‐AMPK (Ser485) regardless of SRT1720 treatment in VSMCs (Fig. 1E). Contrary to previously reported results, we found that H‐89 or forskolin regulated the expression level of p‐AMPK (Ser485) (Fig. 1F). In the previous study, it has been reported that SIRT1 interacts with the cAMP–PKA pathway [33] and ameliorates ADR‐induced VSMC senescence by upregulating the cAMP–PKA/p‐AMPK (Ser485) signaling pathway. However, it is unclear whether SRT1720 can regulate the AKT pathway in ADR‐induced VSMC senescence.

In conclusion, it will be important to investigate the mechanisms by which they interact and the consequences of their cross‐regulations under senescence‐reducing conditions in VSMCs. This study shows that the activities of SIRT1 and p‐AMPK (Ser485) may be involved in the reduction of VSMC senescence. Ultimately, pharmacological activators induced by SIRT1 and p‐AMPK (Ser485) regulate through activation of the cAMP–PKA pathway and alleviate VSMC senescence. Therefore, further studies would be necessary to elucidate the role of cellular senescence.

## Conflict of interest

The authors declare no conflict of interest.

## Author contributions

JYS performed the main experiments, prepared figures and wrote the manuscript. JYS and SGK organized data. JYS and HCC designed the experiments. DHC, J‐RK and HCC supervised this study.
